# Dostarlimab: The miracle drug for the treatment of colorectal cancer

**DOI:** 10.1016/j.amsu.2022.104493

**Published:** 2022-08-27

**Authors:** Pollob Ahmed Shuvo, Anika Tahsin, Md. Mominur Rahman, Talha Bin Emran

**Affiliations:** Department of Pharmacy, Faculty of Allied Health Sciences, Daffodil International University, Dhaka, 1207, Bangladesh; Department of Pharmacy, Faculty of Allied Health Sciences, Daffodil International University, Dhaka, 1207, Bangladesh; Department of Pharmacy, BGC Trust University Bangladesh, Chittagong, 4381, Bangladesh

**Keywords:** Cancer, Colorectal cancer, Dostarlimab, Programmed cell death, Tumors

Dear Editor,

Cancer is nothing but uncontrolled cellular growth. When this uncontrolled cellular growth takes place in colon or rectum area, it is termed as colorectal cancer or simply colon cancer [1]. The epidemiology of colorectal cancer may change drastically depending on geographical locations. It is the second and third most prevalent type of cancer in females and males respectively. Incidence and mortality rates of this type of cancer is significantly lower in females than males [2,3]. Dostarlimab (brand name JEMPERLI) is a collection of laboratory-synthesized molecules which can act as substitute for human antibodies [4]. Dostarlimab is an antibody to anti-programmed cell death receptor-1 (PD-1) which is used for the treatment of adults with mismatch repair-deficient (dMMR) recurrent or advanced solid tumors as well as dMMR-recurrent or advanced cancer in endometrium [5,6].

JEMPERLI attaches to PD-1, found on the surface of T cells. PD-1 in healthy T cells acts as a brake that prevents the cells from starting an uncontrollable immune response. However, PD-1 can inactivate T cells in tumors as well as prevent them from destroying the cancer cells. Cancer cell or normal cell within the tumor mass has an increased concentration of PD-L1 and PD-L2 molecules on their surfaces, which bind to PD-1. When these two molecules (PD-L1 and PD-L2) attach with the PD-1 receptor present on the T cell, the T cell is inactivated and cannot kill the cancer cells. JEMPERLI binds to the PD-1 in a way that resists PD-L1 and PD-L2 from attaching with the PD-1 receptor. This blockade on PD-1 allows the T cells to activate and to attack and kill cancer cells (Fig. 1) [7].

**Fig. 1:**
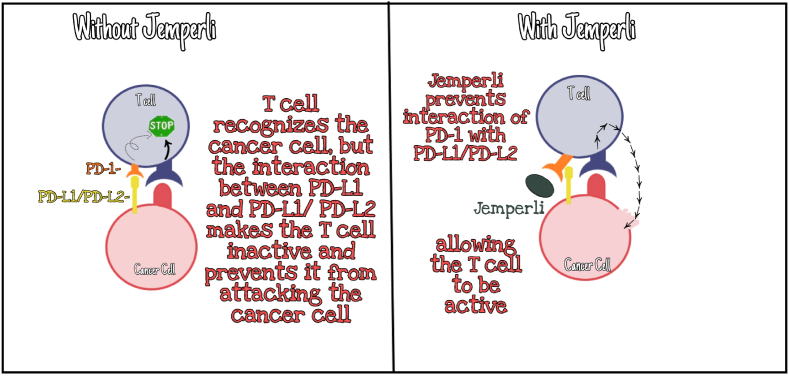
Mechanism of action of JEMPERLI (dostarlimab-gxly).

A small group of colorectal patients (18 individuals) just experienced something no short of a scientific miracle, their disease subsided completely after experimental treatment conducted by a group of doctors at Memorial Sloan Kettering Cancer Center, New York. The trial's result is being labeled as surprising because every patient was cured completely without any exception. These groundbreaking results are unforeseen in cancer research according to some experts [8]. These patients went through treatments like chemotherapy, radiation, and in some cases, life-altering surgery that could alter bowel, urinary and sexual functions. A clinical trial designed by Dr. Diaz in 2017 worked as inspiration for the study. It involved 86 persons who had metastatic cancer that had spread throughout their bodies. However, all of the tumors had a gene mutation that prohibited cells from repairing DNA damage. These mutations are found in 4% of cancer patients. Pembrolizumab, a Merck checkpoint inhibitor, was given to patients in that experiment for up to two years. In around one-third to one-half of the patients, tumors shrunk or stabilized, and they survived longer. Tumors eliminated in 10% of those who took part in the study. The experiment needs to be duplicated in a much larger study, according to the researchers, who point out that the current study only looked at individuals with a unique genetic signature in their tumors. However, they believe that observing total remission in 100% of the patients studied is a highly positive early sign [9].

## Ethical approval

Not applicable.

## Sources of funding

No funding.

## Author contribution

Pollob Ahmed Shuvo: Conceptualization, Data curation, Writing-Original draft preparation, Writing- Reviewing and Editing. Anika Tahsin: Data curation, Writing-Original draft preparation, Writing- Reviewing and Editing. Md. Mominur Rahman: Writing- Reviewing and Editing, Visualization. Talha Bin Emran: Writing- Reviewing and Editing, Visualization, Supervision.

## Registration of research studies

1. Name of the registry: Not applicable.
2. Unique Identifying number or registration ID: Not applicable.
3. Hyperlink to your specific registration (must be publicly accessible and will be checked): Not applicable.

## Guarantor

Talha Bin Emran, Ph.D., Associate Professor, Department of Pharmacy, BGC Trust University Bangladesh, Chittagong 4381, Bangladesh. T: +88-030-3356193, Fax: +88-031-2550224, Cell: +88-01819-942214. https://orcid.org/0000-0003-3188-2272. E-mail: talhabmb@bgctub.ac.bd.

## Consent

Not applicable.

## Data statement

No specific data collected for the above manuscript.

## Provenance and peer review

Not commissioned, internally peer-reviewed.

## Declaration of competing interest

Authors declare that they have no conflicts of interest.
